# Discrepancies in internal and external training load measurements during low-intensity biathlon training

**DOI:** 10.3389/fspor.2024.1455900

**Published:** 2024-09-27

**Authors:** Andreas Kårström, Mikael Swarén, Glenn Björklund

**Affiliations:** ^1^ Department of Health Sciences, Swedish Winter Sport Research Centre, Mid Sweden University, Östersund, Sweden; ^2^ Swedish Biathlon Federation, Östersund, Sweden; ^3^ Swedish Unit of Metrology in Sports, Institution of Health and Welfare, Dalarna University, Falun, Sweden

**Keywords:** adolescent athletes, athlete monitoring, coaching, training organization, TRIMP

## Abstract

**Purpose:**

This study aimed to differentiate external and internal training loads during on-snow biathlon training by adding an accelerometer-derived metric.

**Methods:**

Eleven adolescent athletes were fitted with a combined heart rate (HR) and accelerometer to be worn during all training sessions. Duration, HR, training impulse (TRIMP), and average net force ($AvF_{Net}$) were used as training variables. All training was divided into either low-intensity training (LIT), or high-intensity training (HIT) based on reported intensity. The training was further categorized as training without any shooting practice (NS) or as a combination of skiing and shooting (COMB). Duration, HR, TRIMP, and $AvF_{Net}$ were analyzed in a linear mixed model for the different training modalities.

**Results:**

All training was similar in duration for LIT and HIT sessions (*p* = .0521) and NS and COMB sessions (*p* = .988). TRIMP did not differentiate between LIT or HIT training (*p* = .350) or for NS compared to COMB (*p* = .298). While $AvF_{Net}$ decreased during COMB compared to NS during LIT sessions (*p* < .001) it remained similar during HIT training (*p* = 1.00).

**Conclusion:**

The study’s findings indicated that there were no notable differences in internal training load (TRIMP) when comparing various training intensities and modes. However, the type of training had a significant impact on $AvF_{Net}$, especially leading to a decrease during COMB sessions under LIT conditions. Incorporating an external load metric could offer a fresh approach when prescribing and evaluating training, providing deeper insights into the training load.

## 1 Introduction

Biathlon is an endurance sport that requires both fast cross-country skiing speed and good shooting accuracy (1). A biathlete must, accordingly, train on both parameters for successful performance. Shooting practice can be performed either as a stand-alone exercise or in combination with physical training, executed during both low-intensity training (LIT) and high-intensity training (HIT) (2, 3). Approximately 60% of all endurance training sessions are performed alongside shooting exercises (3). Biathlon physical training consists primarily of various endurance training modes depending on the training phase and access to snow (4, 5). Training modes are mainly divided into sport-specific training (roller skiing and on-snow-skiing, both in the classical and skating technique) and not sport-specific training (e.g., running and cycling).

Heart rate (HR) monitoring is the predominant tool for biathletes to prescribe and monitor training intensity. The methodology is based on the assumptions of a linear relationship to oxygen uptake (6, 7). Furthermore, training intensity is often categorized in predetermined HR training zones based on the percentage of the athlete’s maximal HR (HR_max_) (8). The aim is to accumulate a predetermined training volume in these zones with a supposed link to certain distinguishable metabolic domains. A recent paper (4) showed that successful biathletes accumulated approximately 18% more training volume during upper secondary school as juniors compared to less successful biathletes, with no difference in distribution between different intensity zones. However, HR monitoring as a tool for intensity steering and training quantification has been shown to poorly reflect training intensity during training in undulating or hilly terrains, which are often used as training grounds in biathlon. Several studies have shown that HR poorly reflects instantaneous work during skiing, with HR being highest during downhill skiing (9) or at the beginning of the following section after an uphill (10). HR has also been shown to reflect metabolic demand of various intensities during skiing inadequately (11), and is affected by environmental and psychological factors such as temperature and perceived effort during training (12).

In team sports, wearable accelerometers have been used to quantify the external training load and to improve the profiling of sport-specific demands, such as in ice hockey (13), football (14), and basketball (15). In endurance sports, studies have highlighted the discrepancy between internal intensity and instantaneous work (9, 16, 17) or external training load (18). These results suggest that accelerometer-based metrics may be a valuable tool for further improving the understanding of external training load in endurance sports. Consequently, the external training load for different biathlon training modes has never been investigated. Accelerometry-based metrics could seemingly provide an exciting insight into the multifaceted and intermittent nature of biathlon training. Hence, this exploratory study aimed to add an accelerometer-based metric for differentiating internal and external training loads in different reported training intensities during on-snow biathlon training, with and without shooting exercises, among late adolescent biathletes. It was hypothesized that the external load would be lower than the internal load during training sessions with shooting exercises compared to the training sessions without any shooting exercises.

## 2 Method

### 2.1 Participants

A cohort of eleven adolescent biathletes (male *n* = 7) and (female *n* = 4) age 19 ± 1 years of age at an upper secondary school with a biathlon profile volunteered to participate. All athletes were tier 3 athletes, according to the classification by McKay (17), and accustomed to systematized training and biathlon rifle carriage for at least one year. They received written and oral information about the study and gave their consent by signing an informed consent. The study was approved by the Swedish Ethical Review Authority (Dnr: 202202826-01).

### 2.2 Study design

Every athlete received a combined heart rate and triaxial accelerometer sensor (HR2, Movesense, Vantaa, Finland) and a smartwatch (Tic Watch Pro 3, Mobvoi, Hong Kong, China). The sensor was designed to be worn with a normal HR chest strap. HR and 104 Hz triaxial acceleration were sampled from the sensor to the watch through a smartwatch application (DCS, Kaasa Solutions GmbH, Düsseldorf, Germany). Before its initial use, all athletes performed a maximal running protocol to establish their HR_max_ on a treadmill (Rodby Innovations, Vänge, Sweden). The protocol involved running at a fixed speed (13 km/h for men and 11 km/h for females), with an increase in inclination of 2° for every 2 min, starting at 0°. The test was performed until voluntary termination by either stepping to the side of the treadmill or by signaling to the test leader. The athlete was secured by a safety harness, which was connected to an emergency switch to stop the treadmill in the event of falling. All athletes were instructed to wear the sensor and smart-watch during all their endurance-based training, including both the training at their upper secondary school and their unsupervised training time outside of school hours. The training sessions were categorized based on the reported session type using an online training diary (Maxpulse, Johan Bergman, Östersund, Sweden). The training was categorized as either skiing without shooting [no shooting (NS)] or as a combined shooting session alongside skiing [combination (COMB)]. Exercise intensity was prescribed to the athletes using a five-zone intensity scale (zone 1 55%–72% of HR_max_, zone 2 72%–82% of HR_max_, zone 3 82%–87% of HR_max_, zone 4 87%–92% of HR_max_ and zone 5 92%–100% of HR_max_). However, the training intensity was dichotomized in the present study into two categories. LIT was performed as continuous training within zones 1–2 (55%–82% of HR_max_). All HIT were performed as interval-type sessions in zones 3–5 (>82% of HR_max_). All data were collected over eight weeks (March–April) during the end of the competition phase on snow conditions as skiing only.

### 2.3 Data analyses

Accelerometer data were filtered in Matlab R2022b (Mathworks, Natick, MA, USA) using a fourth-order Butterworth bandpass low-pass filter with 0.1 and 15.0 Hz cut-offs for gravity and noise, respectively (9, 19) The external training load was calculated as the average net force ($AvF_{Net}$) as previously described elsewhere (15), Equation 1.

(1) $$AvF_{Net}=BM\times \frac{\sum_{i=1}^{n}\left(\sqrt{a_{x_{i}}^{2}+a_{y_{i}}^{2}+a_{z_{i}}^{2}}\right)}{n}$$

Where $AvF_{Net}$ is the average net force, BM is the body mass of the biathlete, a_x_, a_y_ and a_z_ are the linear accelerations in the x, y, and z directions, and *n* is the number of samples. In order to determine the effect of different training modalities, the mass of the equipment (skis, rifle, clothing, hydration system etc.) was not included in the biathlete’s $AvF_{Net}$ calculation, as these parameters are subject to changes between sessions and even within a session. A modified training impulse (TRIMP), a mathematical derivation based on HR and duration, was used to calculate the internal training load (20, 21). The modified TRIMP was used because the weighting factor for each training zone in relation to HR, closely reflects the HR zones used in biathlon training. Individual HR response was used to calculate time in each training zone. Time spent below the threshold for zone-1 training was categorized as zone-0 training and was not allocated to LIT training and was therefore calculated as a separate intensity zone.

### 2.4 Statistical analysis

All statistical analyses were made in Jamovi (Jamovi, version 2.2.5, jamovi.org). Normal distribution was checked using Shaprio-Wilk test and by visually checking the residual plot. The only variable to be normally distributed was $AvF_{Net}$. A linear mixed model was used for all variables, due to the statistical robustness of both parametric and non-parametric variables (22). The linear mixed model was employed in a repeated measure design, incorporating both a within-subject factor and a between-group factor. The model was applied to examine the association of shooting factors (NS vs. COMB) and intensity training factors (LIT vs. HIT) in relationship to training duration, internal- and external training loads. Shooting factors and intensity factors were set as fixed factors, and biathletes as random effect, with random intercept across subjects. Each training intensity factor was set as the dependent variable. A new statistical model was made for each of the variables. The significance threshold was established at α < .05. In the instances where the primary interaction demonstrated significance, a post-hoc comparisons were conducted with the Bonferroni correction. Effects size (ES) was calculated as omega square (ω^2^) for all interactions in the analysis. The effect was considered small, medium, and large of values 0.001, 0.06, and 0.14, respectively (23).

$AvF_{Net}\;$data were presented as mean and standard deviation (SD), while non-normally distributed data was presented as median and interquartile range (IQR).

## 3 Results

A total of 82 training sessions and 8,075 minutes of training were collected. Due to some data loss, 79 sessions were included in the study for the analysis of $AvF_{Net}\;$ and 78 sessions were included for TRIMP. Of the total 82 training sessions included in the study, 23% (*n* = 19) of the sessions were performed as HIT, and 24% (*n* = 20) were executed as COMB. There was no difference in session duration between LIT and HIT sessions (103 [86–124] minutes and 93 [88–109] minutes, respectively, *p* = .0521, ES = .0076) or between NS and COMB sessions (100 [89–120] and 96 [86–109] minutes, respectively, *p* = .988, ES = .0130). The distribution of time spent in zone-0 intensity during different training conditions was similar for LIT and HIT sessions (5.5 [1.7–11.8]% and 5.6 [2.3–9.6]%, respectively, *p* = .554, ES = .0084) and NS compared to COMB (5.4 [2.0–12.4]% and 6.9 [2.9–9.2]%, respectively, *p* = .929, ES = .0129). The median duration of each training condition is shown in Table 1.

**Table 1:** Median duration spent in either intensity of zone 0, low-intensity training (LIT), and high-intensity training (HIT) with (COMB) and without (NS) shooting practice.

	LIT-NS	LIT-COMB	HIT-NS	HIT-COMB
Session duration (min)	108 [92–124]	93 [85–105]	92 [87–105]	100 [90–110]
Zone-0 duration (min)	4 [2–15]	6 [4–8]	5 [2–12]	9 [3–10]
LIT duration (min)	85 [58–103]	59 [48–72]	57 [53–60]	59 [49–65]
HIT duration (min)	4 [0–21]	25 [20–33]	29 [18–35]	36 [30–37]

### 3.1 Internal training load

**Figure 1:**
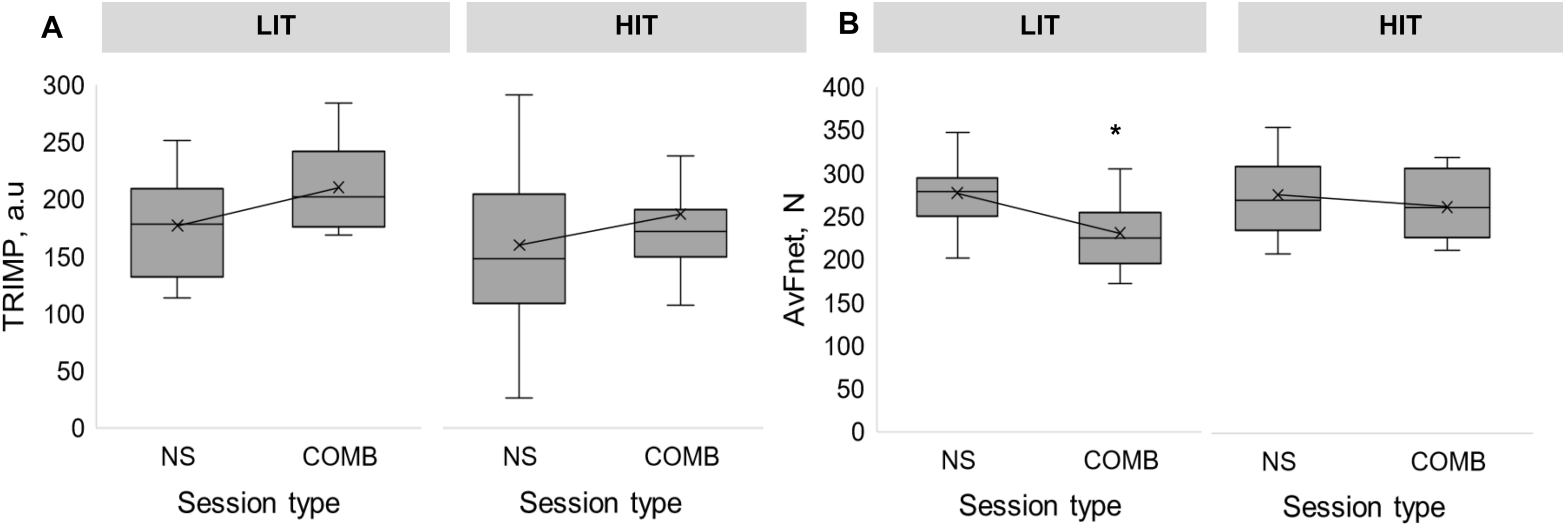
Distribution and median internal **(A)** and external **(B)** load for session type and intensity. *Significant difference between NS and COMB sessions. LIT, Low-intensity training; HIT, High-intensity training; NS, No shooting; COMB, Combination training of physical and shooting practice. Values are presented as median and interquartile range [IQR].

TRIMP did not differentiate between LIT or HIT training (157 [120–202] A.U and 182 [168–222] A.U respectively, *p* = .350, ES = .0016) or for NS sessions compared to COMB (150 [117–205] A.U and 178 [163–216] A.U respectively, *p* = .298, ES = .00052). There was no interaction effect of intensity and shooting variables on TRIMP (*p* = .975, Figure 1).

### 3.2 External training load

There were no differences in external training load between LIT and HIT (267 ± 42 *N* and 271 ± 44 *N*, respectively, *p* = .301, ES = .0010) while $AvF_{Net}\;$ was greater during NS compared to COMB (276 ± 38 *N* and 242 ± 44 *N* respectively, *p* = .015, ES = .0619). While external load decreased during COMB compared to NS during LIT sessions (Figure 1) *p* < .001, ES = .2163) it remained similar during HIT training (*p* = 1.00, ES = .0468, Figure 1).

## 4 Discussion

Based on the lack of knowledge on biathlon training loads, the current study aimed to investigate whether an accelerometer-derived external load measure could be an integrated tool for biathletes to better understand the demands of biathlon on-snow training that are not reflected with HR monitoring. The main finding was that TRIMP remained similar for both LIT and HIT sessions independently of NS or COMB. Further findings of the study were that the external training load during LIT with COMB was lower compared to LIT with NS. This highlights the potential to measure external load as a supplementary metric during on-snow biathlon training sessions.

### 4.1 Internal load and duration

To date, HR monitoring is the primary tool for prescribing and monitoring intensities during biathlon training. The study findings indicate that HR as a tool for evaluating total training load overestimates the training load that a junior biathlon is affected by during on-snow skiing. This is visualized by the difference in external load during LIT sessions, not previously examined in a long-term training setting.

Previous biathlon studies did not show an effect on the HR response during rifle carriage compared to skiing without the rifle in laboratory settings (24, 25) or in an outdoor setting (9). Such data are in line with the findings of the present study, in that a modified TRIMP does not differ between shooting conditions (NS vs. COMB) when the duration of the training sessions is equal. The equal training duration between different training conditions and reported training intensities may indicate that coaches at upper secondary schools are limited by the available training time to balance the school system. Since the school must ensure that student–athletes achieve a sufficient level in both their schooling and sporting performance, there is a delicate balance involved in budgeting the time needed to manage both tasks over the long term. This emphasizes the notion that the sessions are not optimally planned to train on a desired training variable but are rather based on available training time, which is similar between training sessions.

Furthermore, there was no difference in TRIMP between the LIT and HIT sessions, which is potentially explained by the structure of LIT vs. HIT sessions. LIT sessions accumulate a relatively lower HR response over a greater part of the training session before resting (e.g., for drinking or shooting, etc.), while HIT is performed with a greater HR response over a shorter period (usually between 4 and 8 min, depending on the interval session) before a longer rest where the HR is reduced to the LIT zone. However, the data show that even during LIT sessions, HR fluctuates and increases well into the training zones associated with HIT. Previous studies have shown that HR remains elevated when activity alternates between moderate and more intense workloads (26), potentially induced in the present study by the undulating terrain. The result suggests that the ability to differentiate training load based on HR response is not satisfying for on-snow biathlon training.

Different TRIMP models use different mathematical equations to quantify the accumulated training load (21). TRIMP models that use a zone-divided approach justify doing so to gain a more accurate reflection of the account of high-intensity aerobic and/or anaerobic work that may not be shown by the use of average HR or HR-reserve TRIMP methodology. In the present study, a substantial amount of time was recorded in a zone below LIT definitions; zone-0, which would not be considered training intensity and therefore not included in the total TRIMP value. However, more data are needed to show how and when zone-0 time is accumulated; e.g., at what speeds or at which moments during training. Is an athlete considered to be training if their HR corresponds to LIT intensity, even if they are not moving? Conversely, are they not training if they are moving but have an HR response that does not exceed the LIT intensity threshold? The more philosophical question of whether an athlete is undergoing training during zone-0 training should be centered in further research.

### 4.2 External load vs. exercise intensity

The use of an accelerometer-derived metric during training provides insight into biathlon training not visible with HR measures. The data presented showed, for the first time, that during LIT- COMB, junior biathletes accumulate significantly lower external load compared to LIT-NS, even though the training duration is similar. This could potentially be caused by several factors.

Since the accelerometer-derived metric is a result of bodily acceleration and de-acceleration, the major cause of a lower $AvF_{Net\;}$ is likely because of an altered movement during COMB training and the training structure of COMB sessions. Carrying the rifle during skiing has previously been shown to decrease the vertical distance of the upper body while also altering the range of motion in the upper body (27). Previous research also suggests that more force needs to be produced by the lower body instead of the upper body when skiing with the rifle compared to without (25). That factor could explain why the altered movement of the upper body (where the sensor is placed) is impaired, resulting in a lower $AvF_{Net}$. The placement of the sensor should be recognized as a factor in the outcome of this study. One study showed that the $AvF_{Net}\;$ was not different between skiing with or without a rifle during a simulated race when the sensor was placed at the lower spine (9), with less registration of the movement by the upper body. Sensor position must be kept in mind when comparing studies and results using accelerometer data. Furthermore, the training structure could be a potential factor for the lower external load since LIT-COMB often includes more series of shooting drills compared to HIT-COMB. This consequently leads to more time spent standing still while shooting and while refilling ammunition. Implicitly, this would indicate more standing still compared to other types of training sessions, but without compensating by increasing training duration to equal the time spent moving. Coaches should be aware that training administrations could affect the training load’s potential outcome. Future studies should take different training regimes into consideration. The HIT session did not show any differences in external load when comparing NS and COMB, probably due to the similarity in training structure, with similar warm-up, interval- and rest durations.

### 4.3 Limitations

The present study consists of a relatively small sample size. All athletes were attending the same upper secondary school; therefore, it cannot be excluded that data were affected by coaching philosophy and geographical training ground. Finally, no further measurement or variable was included that could explain the differences in external training load between the types of sessions, such as speed or perceived effort.

Future studies are encouraged to include a larger population of biathletes and to sample training variables over a greater range of activities for a more comprehensive understanding of the training load in biathlon training.

### 4.4 Practical implications

The complementary usage of an accelerometer-derived metric during physical training seems to be a valuable tool for highlighting the difference in internal and external training load during on-snow skiing for biathletes. Using an external load metric could provide a new tool for biathlon coaches and biathletes when prescribing and analyzing training. A more comprehensive picture of the total training load allows for future training session adjustments and better training plan cohesion. Athletes and coaches who use TRIMP as the only metric for long-term training load monitoring need to be aware of the uncertainty in the TRIMP method due to the fluctuating HR response during skiing. This proves the difficulty for adolescent athletes to train solely in a prescribed intensity zone during skiing and rather a pragmatic attitude of using a strict HR-based intensity zone is more appropriate.

## 5 Conclusion

In conclusion, the study found no significant differences in internal training load (TRIMP) across different training intensities and modalities. However, external training load ($AvF_{Net}$) was significantly influenced by the type of training, particularly showing a reduction during combined sessions under LIT conditions. These findings suggest that while internal load remains stable, external load is more sensitive to the combination of training modalities, emphasizing the need to consider both internal and external metrics when designing and evaluating training programs.

## Data Availability

The raw data supporting the conclusions of this article will be made available by the authors, without undue reservation.
